# Antikoagulation bei „coronavirus disease 2019“ (COVID-19) – Gesichertes und Kontroverses

**DOI:** 10.1007/s00108-022-01296-x

**Published:** 2022-03-15

**Authors:** Ursula Rauch-Kröhnert, Hanno Riess

**Affiliations:** 1 Klinik für Kardiologie, Campus Benjamin Franklin, Charité – Universitätsmedizin Berlin, Hindenburgdamm 30, 12200 Berlin, Deutschland; 2 Medizinische Klinik m. S. Hämatologie, Onkologie und Tumorimmunologie, Campus Charité Mitte, Charité – Universitätsmedizin Berlin, Charitéplatz 1, 10117 Berlin, Deutschland

**Keywords:** COVID-19/Prognose, Tiefe Venenthrombose, Mikrothrombose, Heparin, Fondaparinux, COVID-19/prognosis, Venous thrombosis, Microthrombosis, Heparin, Fondaparinux

## Abstract

Die Infektion mit dem „severe acute respiratory syndrome coronavirus 2“ (SARS-CoV-2) ist mit einem hohen Risiko mikrovaskulärer Immunthrombosen sowie symptomatischer und inzidenteller Thromboembolien vorrangig im venösen, aber auch im arteriellen System vergesellschaftet. Dies begründet unter anderem die hohe kardiovaskuläre Morbidität und Mortalität der Patienten. Der heutige Kenntnisstand zur Pathophysiologie der Immunthrombosen und zu derzeitigen Strategien der Antikoagulation bei an „coronavirus disease 2019“ (COVID-19) erkrankten Patienten wird in diesem Beitrag zusammenfassend beleuchtet. Gemäß den aktuellen Leitlinien sollen moderat bis schwer erkrankte Infizierte, die sich in stationärer Krankenhausbehandlung befinden, frühzeitig eine Thromboseprophylaxe mit niedermolekularem oder unfraktioniertem Heparin oder alternativ mit Fondaparinux erhalten, sofern kein deutlich erhöhtes Blutungsrisiko vorliegt. Außerhalb der etablierten Therapieindikationen sollte eine intensivierte oder therapeutisch dosierte Prophylaxe auch aufgrund vermehrter Blutungskomplikationen bei diesen kritisch erkrankten Patienten sehr zurückhaltend erwogen werden. Die routinemäßige Fortführung einer prophylaktischen Antikoagulation nach der Krankenhausentlassung wird derzeit nicht empfohlen.

## Abstract (en)

Infection with severe acute respiratory syndrome coronavirus 2 (SARS-CoV-2) is associated with a high risk of microvascular immunothrombosis as well as symptomatic and incidental thromboembolisms, predominantly in the venous system but also in the arterial system. This explains among other things the high cardiovascular morbidity and mortality of the patients. The present state of knowledge on the pathophysiology of immunothrombosis and the strategies of anticoagulation in patients with coronavirus disease 2019 (COVID-19) are summarized and illuminated in this article. According to the current guidelines moderately to severely ill patients who are being treated in hospital should receive thrombosis prophylaxis with low molecular weight or unfractionated heparin or alternatively with fondaparinux, as long as there is no clearly increased risk of bleeding. Apart from the established indications for treatment, an intensified or therapeutic dose prophylaxis should be considered very cautiously in these critically ill patients, also due to the increased bleeding complications. The routine continuation of prophylactic anticoagulation after discharge from hospital is currently not recommended.

Die durch das „severe acute respiratory syndrome coronavirus 2“ (SARS-CoV-2) bedingte Coronavirus-disease-2019(COVID-19)-Pandemie ist mit einer hohen kardiovaskulären Morbidität und Mortalität assoziiert [1, 2]. Die Mehrzahl der moderat bis schwer erkrankten Infizierten muss stationär aufgenommen werden. Mit dem Schweregrad der Erkrankung steigt das Risiko prognosemitbestimmender thromboembolischer und kardiovaskulärer Komplikationen [3–6]. Sie betreffen vorwiegend das venöse, aber auch das arterielle Gefäßsystem und manifestieren sich in unterschiedlichen Organen [7]. Die vorliegende Übersichtsarbeit fasst den gegenwärtigen Kenntnisstand zu Pathophysiologie, klinischer Bedeutung und Antikoagulationstherapie der COVID-19-assoziierten thromboembolischen Erkrankungen zusammen.

## Pathophysiologie der Thrombogenese bei COVID-19

Für den Eintritt in die Wirtszellen bindet SARS-CoV‑2 an den körpereigenen Angiotensin-converting-enzyme-2(ACE2)-Rezeptor [8], der insbesondere auch auf Endothelien und alveolären Epithelzellen stark exprimiert ist. Durch intrazelluläre Replikation und Exozytose des Virus in die Blutstrombahn werden Schädigungen von Alveolarzellen und Endothelien verursacht. Diese führen pulmonal zu entzündlichen Infiltraten, Thromben in der Mikrozirkulation und einer starken systemischen Akute-Phase-Reaktion, oft mit überschießender Produktion proinflammatorischer Zytokine sowie prothrombotischer Aktivierung des Hämostasesystems [9–11].

Bedingt durch die oben genannten Mechanismen äußern sich die thrombotischen Manifestationen bei COVID-19 nicht nur als klassische tiefe Venenthrombose (TVT) oder Lungenembolie (LE) – zusammengefasst als venöse Thromboembolie (VTE) –, sondern auch als Mikrothrombosen in vielen verschiedenen Geweben und Organen [12, 13] mit Ähnlichkeiten zur prothrombotischen disseminierten intravasalen Gerinnung („disseminated intravascular coagulation“ [DIC]), wobei die reaktive Fibrinolyse unter anderem zum Anstieg der D‑Dimere führt.

Laborchemisch sind bei Patienten mit COVID-19 viele Zytokine und Chemokine erhöht, darunter Interleukin(IL)-1, IL‑2 und IL‑6 sowie Tumor-Nekrose-Faktor‑α (TNF-α) und Interferon‑γ (IFN‑γ; [14, 15]). IL‑6 beispielweise bewirkt eine Steigerung der Produktion und Aktivität der Thrombozyten, erhöht die Expression des „tissue factor“ auf Endothelzellen und Monozyten und kann eine endotheliale Dysfunktion auslösen. IFN‑γ hat ähnliche prothrombotische Wirkungen. IL‑2 vermindert durch Hochregulation des Plasminogenaktivatorinhibitors 1 (PAI-1) die Fibrinolyse [14].

Es besteht eine prokoagulatorisch bidirektionale Stimulation vieler Entzündungsmediatoren und der plasmatischen Gerinnung [16]. Dies erklärt, warum der Höhe der D‑Dimer-Werte ähnliche prognostische Bedeutung zukommt wie der Konzentration inflammatorischer Zytokine, beispielsweise des IL‑6; beides etablierte Risikomarker für einen schweren Krankheitsverlauf bei Patienten mit COVID-19 [1, 17–19].

Für Patienten mit COVID-19 wird bereits bei stationärer Aufnahme eine D‑Dimer-Bestimmung empfohlen

In einer multizentrischen retrospektiven Kohortenstudie wurde gezeigt, dass D‑Dimer-Spiegel von mehr als 1000 µg/l mit einer deutlich gesteigerten Mortalität im Krankenhaus assoziiert waren (Odds Ratio [OR] 18,42; 95 %-Konfidenzintervall [KI] 2,64–128,55; *p* = 0,0033; [20]). Der Cut-off-Wert für D‑Dimer von 1500 μg/l besitzt zur Vorhersage von VTE eine Sensitivität und Spezifität von 85 % bzw. 88,5 % und einen negativen prädiktiven Wert von 94,7 % [21]. Daher empfehlen die aktuellen S3-Leitlinien zur Behandlung von Patienten mit COVID-19 bereits bei stationärer Aufnahme eine Bestimmung dieses Markers sowie engmaschige Verlaufsmessungen [22]. Bei schwerer erkrankten Patienten wird zusätzlich ein Monitoring weiterer Hämostaseparameter gefordert (Thrombozytenzahl, Quick/International Normalized Ratio, aktivierte partielle Thromboplastinzeit [aPTT], Fibrinogen, Antithrombin; [22]). Der aus diesen Parametern ableitbare DIC-Score der International Society on Thrombosis and Haemostasis (ISTH) scheint ein weiteres hilfreiches Instrument zur Charakterisierung der COVID-19-assoziierten Koagulopathie (CAC; [21]) bzw. der pulmonalen intravaskulären Koagulopathie (PIC; [23]) sowie zur Prognosebeurteilung zu sein [19].

## Klinische Bedeutung der COVID-19-assoziierten thrombotischen Ereignisse

Aufgrund unterschiedlicher Methoden und der Heterogenität der untersuchten Patientenkollektive ist die genaue Inzidenz der Makro-VTE bei hospitalisierten Patienten mit COVID-19 nicht bekannt. Zwei kürzlich durchgeführte Metaanalysen [24, 25] mit 22.570 bzw. 64.503 stationären COVID-19-Patienten ergaben VTE-Inzidenzen von 38 % bzw. 28 % bei Intensivpatienten und von 17 % bzw. 7 % bei Patienten auf Nichtintensivstationen [26]. Die Inzidenz der tiefen Beinvenenthrombose wird mit 22 % bei Intensivpatienten und 13 % bei Nichtintensivpatienten beziffert; die der LE mit 22 % bzw. 13 % [24]. Die Prävalenz der VTE bei Intensivpatienten betrug jedoch fast 50 % und war somit deutlich höher als die oben genannte, wenn die Patienten systematisch auf das Vorhandensein einer VTE untersucht wurden [25]. Eine ISTH-DIC fand sich bei 71 % der COVID-19-Intensivpatienten, die die Infektion nicht überlebten; die COVID-19-Überlebenden waren davon kaum betroffen [19]. Multiple pulmonale Makro- und Mikrothrombosen zeigten sich auch in den Autopsien von Patienten, die an einem COVID-19-assoziierten Lungenversagen verstorben waren [26, 27]. Weiterhin fanden sich diffuse alveoläre Ödeme und hyaline Membranen, ähnlich wie beim „acute respiratory distress syndrome“ (ARDS), charakterisiert als „microvascular COVID-19 lung vessels obstructive thromboinflammatory syndrome“ (MicroCLOTS; [28]).

Im Gegensatz zu den hohen VTE-Raten bei hospitalisierten Patienten scheinen VTE bei ambulant zu betreuenden Patienten mit COVID-19 nicht häufiger als bei ähnlich schwer erkrankten Patienten ohne COVID-19 zu sein [29, 30].

Neben venösen treten bei schwer an COVID-19 erkrankten Patienten auch arterielle Komplikationen gehäuft auf. Klok et al. berichteten, dass 3 % der an COVID-19 erkrankten Patienten einen Schlaganfall und 1 % arterielle Embolien entwickelten [31]. Auch ein erhöhtes Risiko des Auftretens akuter Koronarsyndrome wird berichtet, meist bei vorbestehender Atherosklerose [32, 33].

## Antikoagulation bei hospitalisierten Patienten mit COVID-19

Aufgrund des bei stationären Patienten mit COVID-19 deutlich erhöhten Risikos hyposymptomatischer VTE (sogenannte inzidentelle VTE; [25, 34]) ist die Indikationsstellung für entsprechende Screeninguntersuchungen großzügig zu stellen (Computertomographie des Thorax bzw. Ultraschalluntersuchung der tiefen Bein- und Beckenvenen). Bei nachgewiesener inzidenteller VTE erfolgt die Therapie analog zu symptomatischen Ereignissen [35]. Idealerweise wäre bei Studien zur primären VTE-Prophylaxe der initiale Ausschluss von inzidentellen VTE vor der Randomisierung zu fordern. Dass dies in den bisher vorliegenden Untersuchungen nicht regelhaft erfolgte, ist bei der Ergebnisbewertung zu berücksichtigen.

### Primärprophylaxe venöser Thromboembolien

Prospektive, randomisierte klinische Studien belegen die hochsignifikante Wirksamkeit der medikamentösen VTE-Prophylaxe bei stationären Patienten mit akuten internistischen Erkrankungen, ohne Zunahme schwerer Blutungen [35]. Unter Berücksichtigung des Vorliegens von Kontraindikationen können diese Daten auch auf nichtintensivpflichtige Patienten mit COVID-19 übertragen werden. In einer ersten großen retrospektiven Beobachtungsstudie aus China, die auch die prognostische Aussagekraft des D‑Dimer-Werts bestätigt, wurde bei stationären COVID-19-Patienten eine reduzierte 28-Tages-Mortalität unter Prophylaxe mit niedermolekularem Heparin (NMH) oder unfraktioniertem Heparin (UFH) nachgewiesen [36]. Die 28-Tages-Mortalität war bei Patienten mit einem mehr als 6‑fach erhöhten D‑Dimer-Wert signifikant erhöht (52,4 % vs. 32,8 %; *p* = 0,017; [36]). Eine weitere retrospektive Auswertung von 4297 Patienten mit COVID-19 zeigt darüber hinaus, dass eine innerhalb von 24 h nach stationärer Aufnahme eingeleitete prophylaktische Antikoagulation gegenüber keiner oder einer später initiierten Antikoagulation die kumulative 30-Tages-Mortalität von 18,7 % (95 %-KI 15,1–22,9 %) auf 14,3 % (95 %-KI 13,1–15,5 %) senkt [37]. Diese Daten belegen die entscheidende Bedeutung der prophylaktischen Antikoagulation für die Prognose auch von hospitalisierten Patienten mit COVID-19. Dabei sollte eine für den Hochrisikobereich zugelassene Dosierung angewendet werden.

Kontrovers wird der Nutzen einer dosisintensivierten, häufig halbtherapeutischen NMH-Dosierung in der Literatur diskutiert [38]. In einer systematischen Metaanalyse von vorwiegend retrospektiven Beobachtungsstudien war die VTE-Rate unter intermediär dosierter Antikoagulation jedoch nicht niedriger als unter einer Hochrisikoprophylaxedosierung [38]. Auch in einer prospektiven, randomisierten Studie an 562 Intensivpatienten zeigte die halbtherapeutische NMH- gegenüber der Standarddosierung keine Vorteile bezüglich des kombinierten Wirksamkeitsendpunkts aus venösen oder arteriellen Thromboembolien, Notwendigkeit einer extrakorporalen Membranoxygenierung und 30-Tages-Mortalität (Ereignisrate 45,7 % vs. 44,1 %; OR 1,06; 95 %-KI 0,76–1,48; *p* = 0,70; [39]). Bei vergleichbarer VTE-Rate (3,3 % vs. 3,5 %) waren klinisch relevante Blutungen mit 6,2 % vs. 3,1 % numerisch häufiger unter der intensivierten Antikoagulation (OR 2,02; 95 %-KI 0,89–4,61; *p* = 0,08). Eine randomisierte Studie an 176 Patienten fand ebenfalls keinen Vorteil einer halbtherapeutischen Enoxaparindosis bezüglich Thromboembolierate, Blutungsereignissen oder 30-Tages-Sterblichkeit [40].

Hochrisikoprophylaxedosierungsempfehlung bei nichtintensivpflichtigen COVID-19-Patienten ist zu unterstützen

Zusammenfassend ist die Empfehlung einer Hochrisikoprophylaxedosierung bei nichtintensivpflichtigen Patienten mit COVID-19 vorbehaltlos zu unterstützen [22]. Bei Patienten mit deutlich erhöhten D‑Dimeren und/oder Entzündungsparametern oder Intensivpflicht sollte die Indikation zu einem VTE-Screening großzügig gestellt werden. Auch bei fehlendem Nachweis einer VTE kann es nach sorgfältiger Abwägung des Blutungsrisikos in Fällen mit zusätzlichen Risikofaktoren, etwa bei einer ausgeprägten Adipositas oder einer Vorgeschichte mit VTE, gerechtfertigt sein, eine intensivierte Prophylaxe mit NMH durchzuführen [22].

### Therapeutische Antikoagulation zur Beeinflussung der Krankheitsprogression

In Anbetracht des fast pathognomonischen Nachweises von Mikrothromben in der pulmonalen Endstrombahn und der hohen Inzidenz von Makrothrombosen bei schwer erkrankten COVID-19-Patienten wurde in einer Vielzahl von klinischen Studien der Effekt einer therapeutischen Antikoagulation auf Gesamtletalität und Notwendigkeit organunterstützender Maßnahmen, wie der invasiven Beatmung, evaluiert [41–47]. Dabei wurden verschiedene Patientengruppen untersucht, beispielsweise ausschließlich auf der Intensivstation behandelte Patienten mit mechanischer Beatmung [45, 48], gemischte Patientengruppen mit unterschiedlichem Bedarf an pulmonaler Unterstützung [47] oder überwiegend nichtintensivpflichtige Patienten mit COVID-19 ohne Atemunterstützung oder Katecholamingabe [46].

#### Nichtintensivpflichtige Patienten

Der Nutzen einer therapeutischen vs. prophylaktischen Antikoagulation mit NMH wurde von den ATTACC-, ACTIV-4a- und REMAP-CAP-Studiengruppen prospektiv und randomisiert untersucht, wobei die Daten der hospitalisierten nichtintensivpflichtigen COVID-19-Patienten getrennt vom Pool der intensivpflichtigen Patienten ausgewertet wurden [46, 53]. Die therapeutische Antikoagulation steigerte das Krankenhausüberleben ohne Organunterstützung um 4 %, wenn die Patienten bei Studieneinschluss keine ventilatorische Unterstützung oder Anwendung von inotropen Medikamenten auf der Intensivstation benötigten, und dies sowohl bei Patienten mit niedrigen als auch bei Patienten mit hohen D‑Dimer-Spiegeln, wobei der Behandlungsnutzen bei Patienten mit höheren D‑Dimer-Spiegeln größer war [46].

Die ACTION-Studie untersuchte den Nutzen des Faktor-Xa-Inhibitors Rivaroxaban bei 615 Patienten mit moderater COVID-19-Erkrankung [47]. Über 30 Tage wurde Rivaroxaban in einer therapeutischen Dosierung von 20 mg (oder 15 mg) im Vergleich zu Enoxaparin (oder UFH) in prophylaktischer Dosis jeweils 1‑mal täglich verabreicht [47]. Bei instabilen Patienten der Rivaroxabangruppe war auch die Gabe von Heparinen in therapeutischer Dosierung erlaubt. Die therapeutische Antikoagulation veränderte die Sterblichkeit oder die Dauer des Krankenhausaufenthalts nicht, führte aber zu einem signifikanten Anstieg schwerer und klinisch relevanter nichtschwerer Blutungen von 2 auf 8 %. Weitere Studien, die direkte orale Antikoagulanzien bei Patienten mit COVID-19 analysieren, befinden sich derzeit in der Abschlussphase [49, 50].

Eine therapeutische Antikoagulation mit NMH oder UFH sollte bei nichtintensivpflichtigen Patienten mit niedrigem Blutungsrisiko und erhöhten D‑Dimer-Konzentrationen (> 2,0 mg/dl) erwogen werden [22]. Wird die Indikation zur therapeutischen Antikoagulation gestellt, ist bei deutlich eingeschränkter Nierenfunktion (errechnete glomeruläre Filtrationsrate < 30 ml/min) ein UFH gegenüber einem NMH zu bevorzugen. Die regelmäßige Bestimmung der Anti-Faktor-Xa-Aktivität erlaubt ein Monitoring der UFH-Therapie auch bei vorbestehend pathologischen Verlängerungen der aPTT im Rahmen einer Koagulopathie oder bei fehlender Verlängerung der aPTT trotz therapeutischer Dosen (UFH-Resistenz), alternativ kann Argatroban in Betracht gezogen werden [51, 52].

#### Intensivpflichtige Patienten

Eine Studie mit 1091 Patienten zeigte, dass eine therapeutische Antikoagulation den primären kombinierten Endpunkt aus thromboembolischen Ereignissen und Sterblichkeit bis Tag 28 nicht beeinflusst (relatives Risiko [RR] 0,99; 95 %-KI 0,86–1,14; [48]). Dagegen war das Risiko der schweren Blutungen nominell erhöht.

In der ACTION-Studie zu Rivaroxaban [47], in der nur 91 Patienten mit schwerem COVID-19-Verlauf untersucht wurden, ergaben sich ähnliche Effekte wie in der REMAP-CAP-Studie [53] zu den intensivpflichtigen Patienten mit COVID-19 im Hinblick auf das Auftreten thrombotischer Ereignisse oder die Sterblichkeit bis Tag 28 (RR 1,03; 95 %-KI 0,70–1,50) sowie hinsichtlich des Auftretens schwerer Blutungen (RR 2,45; 95 %-KI 0,78–7,73; [22]).

Ohne etablierte Indikation ist bei intensivpflichtigen COVID-19-Patienten von Vollantikoagulation abzuratenDie Aufnahme von intensivpflichtigen Patienten in die Studien REMAP-CAP, ACTIV-4a und ATTACC, welche die Vollantikoagulation bei COVID-19 untersuchten, musste vorzeitig gestoppt werden, denn es zeigte sich, dass zwar vermehrte Blutungskomplikationen, aber keine Prognoseverbesserung im Vergleich zu einer Prophylaxedosierung auftraten. Somit ist außerhalb der etablierten Indikationen bei intensivpflichtigen Patienten mit COVID-19 von einer Vollantikoagulation abzuraten [22, 54].

## Antikoagulation bei ambulanten Patienten mit COVID-19

### Antikoagulation bei primär ambulanten Patienten

Die Rekrutierung primär ambulant betreuter Patienten mit COVID-19 in die ACTIV-4b-Studie wurde vorzeitig abgebrochen, da sich kein Signal für den Nutzen einer prophylaktischen oder therapeutischen Antikoagulation oder einer thrombozytenfunktionshemmenden Medikation ergab [55].

### Antikoagulation nach der Krankenhausentlassung

Die routinemäßige Fortführung einer Antikoagulation nach der Krankenhausentlassung wird nicht empfohlen [22, 54], fehlten dazu doch bis vor Kurzem prospektive, randomisierte Studiendaten. Eine aktuelle Studie an 320 COVID-19-Patienten mit bei Entlassung erhöhtem VTE-Risiko zeigt ohne Zunahme von Blutungskomplikationen den Vorteil einer 35-tägigen VTE-Prophylaxe mit 10 mg Rivaroxaban (zurzeit dafür „off label“) im Vergleich zu keiner verlängerten Thromboprophylaxe [56]. Es wurde der IMPROVE-VTE-Score [57] zur VTE-Risiko-Beurteilung verwendet. Der kombinierte Wirksamkeitsendpunkt aus symptomatischen und inzidentellen VTE, symptomatischen arteriellen Ereignissen sowie kardiovaskulärem Tod bis Tag 35 wurde von 9 auf 3 % signifikant reduziert. Im Einzelfall kann somit bei niedrigem Blutungsrisiko und fortbestehend hohem VTE-Risiko, etwa bei deutlich eingeschränkter Mobilität, eine prophylaktische Antikoagulation begründet sein [58]. Bei Patienten, bei denen im Rahmen ihrer COVID-19-Erkrankung eine symptomatische oder inzidentelle VTE nachgewiesen wurde, ist eine Weiterführung der therapeutischen Antikoagulation poststationär gemäß den aktuellen Leitlinien für mindestens 3 Monate erforderlich [59, 60].

**Figure 1:**
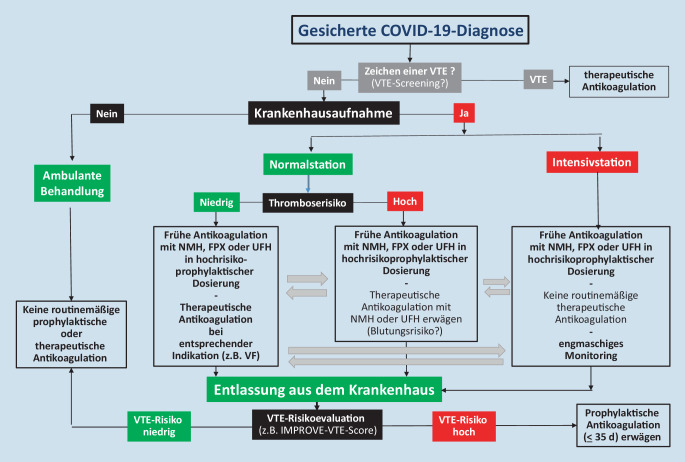

In Abb. 1 ist ein Algorithmus für die Antikoagulation bei Patienten mit COVID-19-Diagnose dargestellt.

## Fazit für die Praxis

- Die „coronavirus disease 2019“ (COVID-19) ist mit einem hohen Risiko mikrovaskulärer Thrombosen sowie symptomatischer und inzidenteller Thromboembolien – vorrangig im venösen, aber auch im arteriellen System – vergesellschaftet.
- Die Indikation zur Ausschlussdiagnostik einer venösen Thromboembolie (Sonographie der Beinvenen, Computertomographie der Lunge) sollte großzügig gestellt werden, insbesondere bei pathologisch erhöhten D‑Dimer-Werten.
- Bei ambulant zu betreuenden Patienten mit COVID-19 wird von einer routinemäßigen antithrombotischen Prophylaxe abgeraten.
- Bei stationärer Aufnahme von Patienten mit Severe-acute-respiratory-syndrome-coronavirus-2(SARS-CoV-2)-Nachweis sollen alle Patienten frühzeitig eine Thromboseprophylaxe mit niedermolekularem oder unfraktioniertem Heparin, alternativ mit Fondaparinux erhalten, sofern kein deutlich erhöhtes Blutungsrisiko vorliegt.
- Auch bei intensivpflichtigen Patienten mit COVID-19 sollte – außerhalb der etablierten Indikationen für eine Vollantikoagulation – routinemäßig eine prophylaktische Antikoagulation erfolgen.
- Bei derzeit ungeklärtem Stellenwert der direkten oralen Antikoagulanzien ist von deren Anwendung im Rahmen einer COVID-19-assoziierten Hospitalisierung abzuraten.
- Die routinemäßige Fortführung einer prophylaktischen Antikoagulation nach der Krankenhausentlassung wird nicht empfohlen.

## References

[1] Huang C, Wang Y, Li X (2020). Clinical features of patients infected with 2019 novel coronavirus in Wuhan, China. Lancet.

[2] Zhou F, Yu T, Du R (2020). Clinical course and risk factors for mortality of adult inpatients with COVID-19 in Wuhan, China: a retrospective cohort study. Lancet.

[3] Perlman S (2020). Another decade, another Coronavirus. N Engl J Med.

[4] Danzi GB, Loffi M, Galeazzi G (2020). Acute pulmonary embolism and COVID-19 pneumonia: a random association?. Eur Heart J.

[5] Guan WJ, Ni ZY, Hu Y (2020). Clinical characteristics of Coronavirus disease 2019 in China. N Engl J Med.

[6] Bilaloglu S, Aphinyanaphongs Y, Jones S, Iturrate E, Hochman J, Berger JS (2020). Thrombosis in hospitalized patients with COVID-19 in a New York City Health System. JAMA.

[7] Zerwes S, Steinbauer M, Gosslau Y (2021). COVID-19-Infektion – Risiko für thrombembolische Komplikationen. Gefäßchirurgie.

[8] Walls AC, Park YJ, Tortorici MA (2020). Structure, function, and antigenicity of the SARS-coV-2 spike glycoprotein. Cell.

[9] Fang XZ, Wang YX, Xu JQ (2021). Immunothrombosis in acute respiratory dysfunction of COVID-19. Front Immunol.

[10] Thachil J, Tang N, Gando S (2020). ISTH interim guidance on recognition and management of coagulopathy in COVID-19. J Thromb Haemost.

[11] Fajgenbaum DC, June CH (2020). Cytokine storm. N Eng J Med.

[12] Liu Y, Cai J, Wang C (2021). Incidence, prognosis, and laboratory indicators of venous thromboembolism in hospitalized patients with coronavisrus disease 2019: a systematic review and meta-analysis. J Vasc Surg Venous Lymphat Disord.

[13] Labo N, Ohnuki H, Tosato G (2020). Vasculopathy and coagulopathy associated with SARS-CoV-2 infection. Cells.

[14] Du F, Liu B, Zhang S (2021). COVID-19: the role of excessive cytokine release and potential ACE2 down-regulation in promoting hypercoagulable state associated with severe illness. J Thromb Thrombolysis.

[15] Quin Qin C, Zhou L, Hu Z (2020). Dysregulation of immune response patients with COVID-19 in Wuhan, China. Clin Infect Dis.

[16] Chambers RC, Scotton CJ (2012). Coagulation cascade proteinases in lung injury and fibrosis. Proc Am Thorac Soc.

[17] Lippi G, Favaloro EJ (2020). D-dimer is associated with severity of coronavirus disease 2019 (COVID-19): a pooled analysis. Thromb Haemost.

[18] Ranucci M, Balotta A, Dedda UD (2020). The procoagulant pattern of patients with COVID-19 acute respiratory distress syndrome. J Thromb Haemost.

[19] Tang N, Li D, Wang X (2020). Abnormal coagulation parameters are associated with poor prognosis in patients with novel coronavirus pneumonia. J Thromb Haemost.

[20] Zhou F, Yu T, Du R (2020). Clinical course and risk factors for mortality of adult inpatients with COVID-19 in Wuhan, China: a retrospective cohort study. Lancet.

[21] Fogarty H, Townsend L, Cheallaigh NC (2020). COVID19 coagulopathy in Caucasian patients. Br J Haematol.

[22] Kluge S, Janssens U, Welte T (2021). S3-Leitlinie – Empfehlungen zur stationären Therapie von Patienten mit COVID-19.

[23] Brunkhorst FM, Weigand MA, Pletz M (2020). S3-Leitlinie Sepsis – Prävention, Diagnose, Therapie und Nachsorge – Langfassung. Med Klin Intensivmed Notfmed.

[24] Mohamed MFH, Al-Shokri S, Shunnar KM (2021). Prevalence of venous thromboembolism in critically-ill COVID-19 patients: systematic review and meta-analysis. Front Cardiovasc Med.

[25] Jimenez D, Garcia-Sanchez A, Rali P (2021). Incidence of VTE and bleeding among hospitalized patients with coronavirus disease 2019: a systematic review and meta-analysis. Chest.

[26] Liu Y, Cai J, Wang C (2021). Incidence, prognosis, and laboratory indicators of venous thromboembolism in hospitalized patients with coronavisrus disease 2019: a systematic review and meta-analysis. J Vasc Surg Venous Lymphat Disord.

[27] Luo W, Yu H, Gou J, Li X (2020). Clinical pathology of critical patient with novel coronavirus pneumonia (COVID-19).pdf.

[28] Ciceri F, Beretta L, Scandroglio AM (2020). Microvascular COVID-19 lung vessels obstructive thromboinflammatory syndrome (MicroCLOTS): an atypical acute respiratory distress syndrome working hypothesis. Crit Care Resusc.

[29] Douillet D, Riou J, Penaloza A (2021). Risk of symptomatic venous thromboembolism in mild and moderate COVID-19: a comparison of two prospective European cohorts. Thromb Res.

[30] Connors JM, Brooks MM, Sciurba FC (2021). Effect of antithrombotic therapy on clinical outcomes in outpatients with clinically stable symptomatic COVID-19: the ACTIV-4B randomized clinical trial. JAMA.

[31] Klok FA, Kruip M, Van Der Meer NJM (2020). Confirmation of the high cumulative incidence of thrombotic complications in critically ill ICU patients with COVID-19: an updated analysis. Thromb Res.

[32] Lodigiani C, Iapichino G, Carenzo L (2020). Venous and arterial thromboembolic complications in COVID-19 patients admitted to an academic hospital in Milan, Italy. Thromb Res.

[33] Bansal M (2020). Cardiovascular disease and COVID-19. Diabetes Metab Syndr.

[34] Nopp S, Moik F, Jilma B, Pabinger I, Ay C (2020). Risk of venous thromboembolism in patients with COVID-19: a systematic review and meta-analysis. Res Pract Thromb Haemost.

[35] Arbeitsgemeinschaft der Wissenschaftlichen Medizinischen Fachgesellschaften (2015) S3-Leitlinie Prophylaxe der venösen Thromboembolie (VTE). http://www.awmf.org/leitlinien/detail/ll/003-001.html. Zugegriffen: 10.12.2021

[36] Tang N, Bai H, Chen X (2020). Anticoagulant treatment is associated with decreased mortality in severe coronavirus disease 2019 patients with coagulopathy. J Thromb Haemost.

[37] Rentsch CT, Beckman JA, Tomlinson L (2021). Early initiation of prophylactic anticoagulation for prevention of coronavirus disease 2019 mortality in patients admitted to hospital in the United States: cohort study. BMJ.

[38] Patell R, Chiasakul T, Bauer E (2021). Pharmacologic thromboprophylaxis and thrombosis in hospitalized patients with COVID-19: a pooled analysis. Thromb Haemost.

[39] Sadeghipour P, Talasaz AH, Rashidi F (2021). Effect of intermediate-dose vs standard-dose prophylactic anticoagulation on thrombotic events, extracorporeal membrane oxygenation treatment, or mortality among patients with COVID-19 admitted to the intensive care unit: the INSPIRATION randomized clinical trial. JAMA.

[40] Perepu US, Chambers I, Wahab A (2021). Standard prophylactic versus intermediate dose enoxaparin in adults with severe COVID-19: A multi-center, open-label, randomized, controlled trial. J Thromb Haemost.

[41] Bikdeli B, Talasaz AH, Rashidi F (2020). Intermediate versus standard-dose prophylactic anticoagulation and statin therapy versus placebo in critically-ill patients with COVID-19: Rationale and design of the INSPIRATION/ INSPIRATION-S studies. Thromb Res.

[42] Bikdeli B, Madhavan MV, Jimenez D (2020). COVID-19 and thrombotic or thromboembolic disease: implications for prevention, antithrombotic therapy, and follow-up. J Am Coll Cardiol.

[43] Nadkarni GN, Lala A, Bagiella E (2020). Anticoagulation, mortality, bleeding and pathology among patients hospitalized with COVID-19: a single health system study. J Am Coll Cardiol.

[44] Paranjpe I, Fuster V, Lala A (2020). Association of treatment dose anticoagulation with in-hospital survival among hospitalized patients with COVID-19. J. Am. Coll. Cardiol..

[45] Lemos ACB, do Espírito SDA, Salvetti MC (2020). Therapeutic versus prophylactic anticoagulation for severe COVID-19: a randomized phase II clinical trial (HESACOVID). Thromb Res.

[46] Lawler PR, Goligher EC, Berger JS (2021). Therapeutic anticoagulation with heparin in noncritically ill patients with Covid-19. N Engl J Med.

[47] Lopes RD, de Barros ESPGM, Furtado RHM (2021). Therapeutic versus prophylactic anticoagulation for patients admitted to hospital with COVID-19 and elevated D-dimer concentration (ACTION): an open-label, multicentre, randomised, controlled trial. Lancet.

[48] Goligher EC, Bradbury CA, McVerry BJ (2021). Therapeutic anticoagulation with heparin in critically ill patients with Covid-19. N Engl J Med.

[49] Effect of Anticoagulation Therapy on Clinical Outcomes in COVID-19 (COVID-PREVENT). ClinicalTrials.gov Identifier: NCT04416048

[50] FREEDOM COVID-19 Anticoagulation Strategy (FREEDOM COVID). ClinicalTrials.gov Identifier: NCT04512079

[51] Treichl B, Bachler M, Lorenz I (2015). Efficacy of argatroban in critically ill patients with heparin resistance: a retrospective analysis. Semin Thromb Hemost.

[52] McGlynn F, McGrath J, Varghese C (2020). Argatroban for therapeutic anticoagulation for heparin resistance associated with Covid-19 infection. J Thromb Thrombolysis.

[53] The REMAP-CAP, ACTIV-4a, and ATTACC Investigators (2021). Therapeutic anticoagulation in critically ill patients with Covid-19. N Engl J Med.

[54] Fachgruppe COVRIIN am Robert-Koch-Institut (2021) Medikamentöse Therapie bei COVID-19 mit Bewertung durch die Fachgruppe COVRIIN am Robert Koch-Institut. Geschäftsstelle des STAKOB. 10.25646/7743.17. Zugegriffen: 26. Nov. 2021

[55] Connors JM, Brooks MM, Sciurba FC (2021). Effect of antithrombotic therapy on clinical outcomes in outpatients with clinically stable symptomatic COVID-19 the ACTIV-4B randomized clinical trial. JAMA.

[56] Ramacciotti E, Barile Agati L, Calderaro D (2022). Rivaroxaban versus no anticoagulation for post-discharge thromboprophylaxis after hospitalisation for COVID-19 (MICHELLE): an open-label, multicentre, randomised, controlled trial. Lancet.

[57] Spyropoulos AC, Lipardi C, Xu J (2020). Modified IMPROVE VTE risk score and elevated D-dimer identify a high venous thromboembolism risk in acutely ill medical population for extended thromboprophylaxis. TH Open.

[58] Bai C, Chotirmall SH, Rello J (2021). Updated guidance on the management of COVID-19: from an American Thoracic Society/European Respiratory Society coordinated International Task Force. Eur Respir Rev.

[59] [2019] ESC Guidelines for the diagnosis and management of acute pulmonary embolism developed in collaboration with the European Respiratory Society (ERS). European Heart Journal 41:543–603. 10.1093/eurheartj/ ehz405 10.1093/eurheartj/ehz405 31504429

[60] Kearon C, Akl EA, Ornelas J, Blaivas A, Jimenez D, Bounameaux H (2016). Antithrombotic therapy for VTE disease: CHEST guideline and expert panel report. Chest.

